# Mental and psychosocial health among youth after their first psychiatric hospitalization: a feasibility study

**DOI:** 10.1186/s13104-022-06132-x

**Published:** 2022-06-28

**Authors:** Mark A. Ferro, Christy K. Y. Chan, John D. Vanderkooy, Laurie Horricks, Laura Duncan, Ellen L. Lipman

**Affiliations:** 1 School of Public Health Sciences, University of Waterloo, Waterloo, Canada; 2 Child and Adolescent Inpatient Psychiatry, Grand River Hospital, Kitchener, Canada; 3 Child and Youth Mental Health Program, McMaster Children’s Hospital, Hamilton, Canada; 4 Department of Psychiatry and Behavioural Neurosciences, McMaster University, Hamilton, Canada

**Keywords:** Adolescent, Child, Hospitalization, Mental illness, Quality of life

## Abstract

**Objective:**

This pilot study investigated the feasibility of studying 12-month readmission of youth aged 10–16 years following their first psychiatric hospitalization and changes in youth mental and psychosocial health prospectively.

**Results:**

Inpatient youth with a first psychiatric hospitalization and their parents were recruited from a regional hospital in Canada. Data were collected at recruitment, and at 3-, 6-, and 12-months post-discharge. Repeated measures ANOVA was performed to assess changes in health outcomes. Nineteen eligible youth were approached and 15 (78.9%) consented to participate (13.9 ± 2.0 years, 73.3% female). Eleven youth (73.3%) gave permission to contact their parents, all of whom participated (39.2 ± 7.6 years). Four youth dropped out of the study (26.7%) and six youth-parent dyads completed all four follow-ups. The readmission rate was 20.0% (n = 3) over 12 months. Significant changes in youth-reported symptoms of conduct disorder (F = 3.0, p = 0.06) and adverse childhood experiences (F = 3.4, p = 0.05) were found. Changes in parent-reported youth mental health symptoms (F = 3.1, p = 0.06), particularly among internalizing disorders, youth health-related quality of life (F = 11.3, p < 0.01), and youth disability (F = 2.7, p = 0.08) were significant. This preliminary work demonstrates the feasibility of, and need to, engage youth and their families to understand their mental and psychosocial health during this vulnerable period of time.

## Introduction

Approximately 20% of youth have a mental illness, resulting in compounding negative effects on development [1]. Psychiatric hospitalizations and readmissions are common and costly to the health system. Recent data shows increasing rates of youth psychiatric hospitalizations and emergency departments visits for mental health by 60% and 61%, respectively between 2008/09 and 2018/19 [2]. Psychiatric hospitalization often signals a crisis point for youth and their families. Hospitalized youth have substantial emotional distress and lower life quality [3] and families report substantial stress [4]. Effects are pervasive as mental health professionals report burnout [5] and large costs are incurred due to lost productivity within society [6]. Thus, the implications on public health are large [7]. Knowledge of health outcomes following hospitalization or readmission is inadequate [8]. While larger studies using administrative databases to examine readmission rates have been reported, very few have been conducted in Canada, none of which have investigated psychosocial outcomes. Thus, the aim of this pilot study was to assess the feasibility of prospectively studying readmissions and mental and psychosocial health (e.g., quality of life) outcomes among youth experiencing their first psychiatric hospitalization. The study and its design were considered feasible if the following objectives were met: (1) ≥ 50% of eligible youth enlisted into the study; (2) ≥ 50% of enlisted youth were retained during the 12-month follow-up; and, (3) < 20% missing item-level data on completed measures. If feasibility is confirmed, it will provide the impetus for the implementation of a large-scale study of psychiatric readmissions and outcomes associated with readmission to inform preventive interventions for youth with mental illness.

## Main text

### Methods

#### Sample and procedure

Youth experiencing their first psychiatric hospitalization were recruited from inpatient services at a regional hospital in Ontario, Canada between January and February 2020. Eligibility criteria for this study were youth aged 10–16 years who had adequate English skills to complete the in-person interview and mail questionnaires. Youth were excluded if their mental health prevented participation (e.g., risk for harm to others, lack of capacity to consent). In consultation with the clinical manager of the child and adolescent psychiatry inpatient unit, nurses identified eligible youth, introduced the study, and invited youth interested in participating in the study to speak with research staff. Research staff then described the study to youth, obtained informed consent (including an assessment of their decisional capacity) [9], and sought permission from youth to contact their parents/guardians for participation. Youth were eligible to participate without parental participation. All participants (youth and parents) provided informed written consent. Data were collected with researcher-led structured interviews and self-reported questionnaires using lightweight tablets in the hospital. Youth and parent reports were completed for all measures. Follow-up included data collection at three, six, and 12-months post-discharge whereby structured interviews were conducted by phone and questionnaires by mail. Readmission was defined as a hospital admission to any psychiatric inpatient unit or a psychiatric assessment unit in an emergency department during the 12-month follow-up.

#### Measures

The Mini International Neuropsychiatric Interview for Children and Adolescents (MINI-KID) is a validated diagnostic interview for mental illness in youth aged 6–17 years [10] which was administered by trained research staff to youth. It is aligned with the Diagnostic and Statistical Manual of Mental Disorders (DSM-5) and International Statistical Classification of Diseases and Related Health Problems (ICD-10). The most common mental illnesses affecting youth were assessed [1].

The Ontario Child Health Study Emotional Behavioural Scales (OCHS-EBS) is a validated 52-item self-reported checklist measuring youth psychopathology [11, 12]. Items are scored on a three-point response scale, which are summed to total, internalizing, externalizing, and illness-specific scores. Higher scores indicate greater frequency of symptoms.

Youth health-related quality of life (HRQL) was measured using the self-reported KIDSCREEN-27 [13]. KIDSCREEN-27 is a validated scale scored using a five-point Likert scale to assess HRQL across five domains: physical well-being, psychological well-being, autonomy & parent relations, social support & peers, and school environment [14, 15]. T-scores (mean 50, standard deviation 10) are computed with higher scores indicating better HRQL.

The World Health Organization Disability Assessment Schedule (WHODAS) 2.0 is a standardized self-reported instrument for measuring disability [16], which includes 12 items scored using a five-point response scale. Scores are summed with higher scores indicating lower functioning/greater disability. The WHODAS 2.0 has excellent psychometric properties [17].

The 10-item Perceived Stress Scale (PSS) assesses the extent to which an individual perceives aspects of their life as uncontrollable, unpredictable, and overloading [18]. Responses to each item, which target thoughts and feelings from the past month, are based on a five-point Likert scale, with higher scores indicating elevated levels of perceived psychological stress.

Family environment was measured using two scales. The 10-item, self-reported Adverse Childhood Experience Questionnaire (ACE) was used to measure exposure to maltreatment/abuse and household dysfunction in childhood [19, 20]. Responses are binary (no/yes) and higher sum scores suggest greater frequency of previous adverse experiences. Current family functioning was measured using the General Functioning subscale of the McMaster Family Assessment Device (FAD) [21]. The 12 items are based on four-point response scale, which were summed to a total score. Higher scores indicating better overall family functioning.

#### Analyses

Summary statistics were used to describe the sample and outcomes using youth and parent reports. Complete case analysis was conducted on six youth-parent dyads (those that completed all four assessments). Repeated measures ANOVA was performed to assess changes in youth mental and psychosocial outcomes from baseline to 12 months (normality assumption was satisfied and results were consistent with the non-parametric Friedman test). Analyses were performed using IBM SPSS version 28.0 (Armonk, NY: IBM Corp). Hypothesis tests were two-sided and given that this was a feasibility study, type one error was set at α = 0.10.

### Results

#### Sample characteristics

Nineteen eligible youth were approached and 15 (78.9%) provided informed consent. Youth participants had a mean age of 13.9 ± 2.0 years and 73.3% were female. Eleven youth (73.3%) provided permission to contact their parents, all of whom participated (mean age 39.2 ± 7.6 years, 100% female). Five parents (45.5%) were in a partnered relationship, and 54.4% (n = 6) had completed postsecondary education. Most parents (72.7%) reported annual household incomes of < $90,000. Table 1 shows additional characteristics of the sample.

**Table 1:** Baseline characteristics of the full sample and the six youth-parent dyads

Characteristics	Full sample	Youth-parent dyads
Youth*, n*	15	6
Age, years	13.9 ± 2.0	14.3 ± 1.6
Female	11 (73.3)	5 (83.3)
Immigrant	1 (6.7)	0 (0)
Disability, WHODAS 2.0	30.6 ± 6.4	31.2 ± 6.4
Mental health symptoms, OCHS-EBS	40.7 ± 10.4	45.0 ± 10.9
Metal disorder		
Major depressive	13 (86.7)	5 (83.3)
Manic/hypomanic episode	5 (33.3)	3 (50.0)
Separation anxiety	1 (6.7)	1 (16.7)
Social phobia	2 (13.3)	2 (33.3)
Specific phobia	4 (26.7)	2 (33.3)
Obsessive–compulsive	5 (33.3)	3 (50.0)
Generalized anxiety	7 (46.7)	3 (50.0)
Attention-deficit/hyperactivity	5 (33.3)	2 (33.3)
Conduct	2 (13.3)	1 (16.7)
Oppositional defiant	2 (13.3)	1 (16.7)
Parent*, n*	11	6
Age, years	39.2 ± 7.6	43.5 ± 7.7
Female	11 (100)	6 (100)
Immigrant	2 (18.2)	1 (16.7)
Partnered relationship	5 (45.5)	4 (66.7)
Postsecondary graduate	6 (54.4)	1 (16.7)
Household income ≥ $90,000	3 (27.3)	3 (50.0)

Data are reported as mean ± SD or frequency (percent)

The mean length of stay in hospital was 7.0 ± 4.6 days. Suicidality was reported by 93.3% (n = 14) of youth. Major depressive episode and generalized anxiety were the most common mental illnesses, reported by 86.7% (n = 13) and 46.7% (n = 7) of youth, respectively. The mean OCHS-EBS score was 40.7 ± 10.4 and mean WHODAS 2.0 score was 30.6 ± 6.4.

#### Study attrition

Four youth (26.7%) actively withdrew from the study during the 12-month follow-up; two between baseline and three months and two between three and six months. All youth completed the MINI-KID at baseline; completion rates of MINI-KID were 76.9%, 72.7%, and 63.6% at 3, 6, and 12 months, respectively. Completion rates of youth self-reports (OCHS-EBS, KIDSCREEN-27, WHODAS 2.0, PSS, ACE, FAD) were 100% at baseline and 61.5%, 81.8%, and 81.8% at subsequent follow-ups. For parents, completion rates were 90.9%, 72.7%, 88.9%, and 88.9% at baseline, 3, 6, and 12 months. Missing item-level data for youth and parents who completed self-reported questionnaires was < 5% at each time-point. Six youth-parent dyads completed self-reported questionnaires at each time-point. Indicators for study feasibility were met.

#### Readmission events

The psychiatric readmission rate was 20.0% (n = 3; mean age 13.6 ± 1.5 years; 100% female). None of the participants who withdrew had a readmission event. No statistically significant differences in sample characteristics were found between youth who did vs. did not experience readmission. The mean time to readmission was 159.7 ± 125.4 days. One of the youth experienced readmission within one month post-discharge and two youth were readmitted within 6- to 12-month post-discharge. At readmission, two youth and one parent responded to complete the readmission interview and questionnaires. Both youth had major depressive episode and attention-deficit hyperactivity disorder, and both reported suicidality at readmission. These youth reported WHODAS 2.0 scores of 31, had a moderate stress (PSS of 14 and 26), and ACE scores > 4.

#### Mental health and psychosocial outcomes

Psychosocial outcomes for all youth are reported in Table 2. Outcomes for youth-parent dyads that completed all time-points are reported in Table 3. Based on repeated measures ANOVA, parents reported statistically significant decreases in OCHS-EBS total symptoms [F(3,15) = 3.1; p = 0.06] and internalizing symptoms [F(3,15) = 6.4; p = 0.01] of youth mental health. Illness-specific changes were found for parent-reported major depressive [F(3,15) = 7.9; p < 0.01] and separation anxiety disorders [F(3,15) = 3.0; p = 0.06]. In contrast, youth reported a statistically significant increase in OCHS-EBS conduct disorder from baseline to 12 months [F(3,15) = 3.0; p = 0.06].

**Table 2:** Means and standard deviations for youth mental and psychosocial outcome measures at baseline and follow-ups

Measure	Parent report				Youth report			
	Baseline	3 months	6 months	12 months	Baseline	3 months	6 months	12 months
OCHS-EBS, *n*	10	8	8	8	15	8	9	9
OCHS-EBS total	50.7 ± 17.7*	42.0 ± 22.0	44.3 ± 15.5	36.3 ± 20.5	40.7 ± 10.4*	40.4 ± 7.3	43.8 ± 8.9	44.3 ± 13.8
Externalizing disorders	18.2 ± 10.8	18.1 ± 12.2	16.9 ± 8.2	15.9 ± 11.4	15.2 ± 6.0	15.1 ± 4.1	16.1 ± 6.4	17.6 ± 6.9
Conduct	3.3 ± 3.8	4.3 ± 5.1	2.4 ± 2.3	3.6 ± 4.5	2.3 ± 3.2	1.4 ± 1.3	2.9 ± 2.9	3.0 ± 2.4
Oppositional defiant	7.4 ± 4.0*	6.8 ± 4.1	7.4 ± 3.7*	6.1 ± 3.9	4.7 ± 2.9*	4.7 ± 1.8	4.6 ± 1.9*	5.5 ± 2.6
Attention-deficit/hyperactivity	7.5 ± 4.2	7.1 ± 4.1	7.2 ± 3.6	6.1 ± 3.8	8.2 ± 2.0	9.0 ± 2.1	8.6 ± 2.3	9.1 ± 3.2
Internalizing disorders	32.5 ± 9.9*	23.9 ± 10.4	27.4 ± 8.3	20.4 ± 10.3	25.5 ± 8.8*	25.3 ± 5.4	27.7 ± 5.6	26.7 ± 11.3
Major depressive	13.0 ± 2.9*	8.3 ± 3.8	9.5 ± 2.9	6.8 ± 3.9	9.7 ± 4.3*	10.4 ± 3.4	10.4 ± 3.5	9.6 ± 3.2
Generalized anxiety	7.9 ± 2.7	6.8 ± 3.4	7.4 ± 2.7	6.5 ± 3.4	6.9 ± 3.2	6.8 ± 2.2	7.1 ± 2.1	6.6 ± 3.6
Separation anxiety	5.4 ± 4.5	3.6 ± 3.0	3.6 ± 2.9	2.4 ± 2.6	3.3 ± 2.6	2.6 ± 2.0	3.8 ± 3.2	4.3 ± 4.0
Social phobia	6.2 ± 3.3	5.3 ± 2.9	6.9 ± 2.2	4.8 ± 2.8	5.7 ± 2.2	5.6 ± 1.8	6.4 ± 2.2	6.3 ± 2.9
KIDSCREEN-27, *n*	10	8	8	8	15	8	9	9
Physical well-being	28.8 ± 5.3*	35.1 ± 7.5	32.5 ± 9.1	35.1 ± 9.6	34.1 ± 8.2*	35.7 ± 7.6	35.3 ± 5.5	35.0 ± 4.9
Psychological well-being	25.3 ± 7.0*	31.9 ± 5.9*	34.7 ± 8.2	36.1 ± 7.2	33.5 ± 7.5*	38.6 ± 6.6*	35.7 ± 5.8	33.2 ± 4.0^a^
Autonomy & parent relations	39.8 ± 9.0	43.4 ± 5.1	43.7 ± 7.2^a^	42.9 ± 9.7^a^	40 ± 5.4	42.6 ± 5.2	40.4 ± 2.7	44.2 ± 6.5
Social support & peers	32.7 ± 11.6*	36.4 ± 15.9	43.8 ± 8.4	41.2 ± 10.3^a^	44.5 ± 15.9*	41.5 ± 10.6	42.1 ± 10.6^a^	40.4 ± 13.0
School environment	37.3 ± 6.0	37.1 ± 10.7	38.2 ± 15.2^a^	44 ± 18	38.9 ± 8.3	33.2 ± 8.2^a^	37.3 ± 4.8^a^	38.6 ± 7.4
WHODAS 2.0, *n*	10	8	8	7^b^	15	8	9	9
WHODAS 2.0 total	32.8 ± 12.0	27.6 ± 11.3	29.6 ± 6.4	26.7 ± 9.1	30.6 ± 6.4	28.5 ± 6.5	26.3 ± 4.1	33.0 ± 6.3
PSS, *n*	10	8	8	8	15	8	9	8^**c**^
PSS total	22.4 ± 7.4	14.6 ± 6.1*	18.4 ± 6.5*	19.8 ± 9.0	24.5 ± 4.6	21.8 ± 6.8*	22.8 ± 3.5*	24.0 ± 5.4
ACE, *n*	10	8	8	8	15	8	9	9
ACE total	3.1 ± 2.2	2.6 ± 1.2	3.3 ± 2.7	3.1 ± 2.2	3.7 ± 2.5	2.5 ± 1.9	4.2 ± 2.9	4.3 ± 2.8
FAD, *n*	10	8	8	8	15	8	9	8^**d**^
FAD total	2.0 ± 0.4*	2.1 ± 0.5	1.9 ± 0.3	2.1 ± 0.4*	1.6 ± 0.5*	1.8 ± 0.5	1.7 ± 0.3	1.6 ± 0.5*

*Independent t-tests were performed to compare parent- and youth-reported scores at each time-point. Significant level set at p < 0.10

^a^Calculation of KIDSCREEN-27 domain scores was not possible due to > 1 item per domain questions were left unanswered

^b^One parent-reported WHODAS 2.0 was excluded for analysis due to > 1 item were left unanswered

^c^One youth-reported PSS was excluded for analysis due to > 1 item were left unanswered

^d^One youth-reported FAD was excluded for analysis due to > 40% of item were left unanswered

**Table 3:** Descriptive statistics and repeated measures ANOVA for all outcome measures of the six youth-parent dyads

	Parent report							Youth report						
	***N***	Mean ± SD				***F***	***p***	***N***	Mean ± SD				***F***	***p***
		**Baseline**	**3 months**	**6 months**	**12 months**				**Baseline**	**3 months**	**6 months**	**12 months**		
OCHS-EBS total	6	52.0 ± 20.7	44.5 ± 22.6	39.6 ± 15.1	31.6 ± 20.9	3.1	0.06*	6	45.0 ± 10.9	43.1 ± 5.4	41.6 ± 9.6	47.9 ± 12.1	0.7	0.58
Externalizing disorders	6	18.7 ± 9.4	19.3 ± 12.1	15.7 ± 9.3	14.8 ± 12.8	0.7	0.48	6	15.8 ± 4.3	16.5 ± 3.6	15.5 ± 7.3	18.9 ± 6.4	1.4	0.29
Conduct	6	3.0 ± 2.8	5.0 ± 5.6	2.2 ± 2.6	3.7 ± 5.1	1.0	0.42	6	2.0 ± 1.3	1.9 ± 1.2	2.8 ± 3.1	3.6 ± 2.1	3.0	0.06*
Oppositional defiant	6	7.5 ± 3.6	6.8 ± 3.3	6.5 ± 3.9	5.5 ± 4.3	1.2	0.33	6	4.8 ± 2.1	5.2 ± 1.7	4.5 ± 2.3	5.8 ± 2.9	0.6	0.65
Attention-deficit/hyperactivity	6	8.2 ± 3.8	7.5 ± 4.0	7.1 ± 4.2	5.7 ± 4.1	1.0	0.41	6	9.0 ± 1.7	9.5 ± 2.1	8.2 ± 2.7	9.5 ± 3.0	1.1	0.36
Internalizing disorders	6	33.3 ± 11.9	25.2 ± 11.1	23.8 ± 6.0	16.7 ± 8.6	6.4	0.01*	6	29.2 ± 9.3	26.6 ± 4.5	26.2 ± 6.1	29.0 ± 12.6	0.4	0.75
Major depressive	6	13.7 ± 3.1	9.5 ± 3.4	8.5 ± 2.1	6.0 ± 4.3	7.9	< 0.01*	6	11.8 ± 3.9	11.3 ± 2.3	9.3 ± 3.8	9.7 ± 3.6	0.9	0.46
Generalized anxiety	6	8.0 ± 3.3	7.2 ± 3.6	6.2 ± 1.7	5.2 ± 2.8	2.4	0.10	6	7.0 ± 3.0	7.5 ± 1.8	6.5 ± 1.9	7.7 ± 3.1	0.7	0.56
Separation anxiety	6	5.5 ± 5.0	3.3 ± 3.4	2.7 ± 2.7	1.5 ± 2.0	3.0	0.06*	6	4.5 ± 2.8	2.7 ± 2.3	3.8 ± 3.5	5.7 ± 4.2	1.6	0.22
Social phobia	6	6.2 ± 3.8	5.2 ± 3.3	6.5 ± 2.2	4.0 ± 2.1	2.3	0.12	6	5.8 ± 1.7	5.2 ± 1.9	6.5 ± 1.9	6.0 ± 3.0	0.8	0.53
KIDSCREEN-27 domain														
Physical well-being	6	30.1 ± 5.7	34.5 ± 8.4	35.2 ± 8.2	37.7 ± 9.4	2.3	0.12	6	34.6 ± 4.5	33.8 ± 4.0	37.3 ± 5.8	36.9 ± 2.8	1.1	0.39
Psychological well-being	6	23.2 ± 5.9	30.7 ± 6.5	36.3 ± 8.1	38.2 ± 7.1	11.3	< 0.01*	6	32.4 ± 5.2	37.4 ± 6.2	36.6 ± 6.0	32.6 ± 4.2	2.1	0.15
Autonomy & parent relations	6	44.5 ± 7.9	42.3 ± 3.6	45.6 ± 5.8	45.2 ± 8.2	0.7	0.57	6	42.0 ± 7.5	42.7 ± 6.1	40.8 ± 3.4	44.5 ± 5.7	0.8	0.52
Social support & peers	6	32.2 ± 13.9	37.5 ± 16.8	45.6 ± 6.0	41.4 ± 11.3	1.8	0.19	5^a^	44.6 ± 17.4	44.2 ± 10.5	44.3 ± 5.1	41.6 ± 6.7	0.1	0.83
School environment	5^a^	37.2 ± 7.3	33.1 ± 10.7	40.7 ± 17.8	47.7 ± 15.2	2.3	0.13	3^a^	38.7 ± 0.0	33.4 ± 3.1	34.8 ± 2.0	35.4 ± 2.3	–^b^	
WHODAS 2.0 total	6	34.3 ± 12.0	31.5 ± 10.0	27.8 ± 6.5	24.3 ± 7.1	2.7	0.08*	6	31.2 ± 6.4	28.0 ± 6.9	25.3 ± 4.1	31.0 ± 5.9	1.3	0.32
PSS total	6	20.2 ± 6.6	14.7 ± 4.8	16.3 ± 5.6	17.2 ± 9.0	1.8	0.19	6	26.2 ± 4.1	23.5 ± 5.8	22.0 ± 3.0	24.2 ± 6.4	0.8	0.53
ACE total	6	2.5 ± 1.5	2.7 ± 1.4	2.0 ± 1.7	2.2 ± 1.5	1.0	0.42	6	2.2 ± 2.0	2.7 ± 2.2	3.2 ± 2.9	3.5 ± 2.7	3.4	0.05*
FAD total	6	2.0 ± 0.5	2.0 ± 0.5	1.8 ± 0.1	2.0 ± 0.4	1.2	0.34	6	1.6 ± 0.7	1.7 ± 0.5	1.7 ± 0.3	1.6 ± 0.5	0.1	0.87

^*^Significant level set at p < 0.10

^a^Calculation of KIDSCREEN-27 domain scores was not possible due to > 1 item per domain questions were left unanswered

^b^No repeated measures ANOVA was performed due to the small sample number of KIDSCREEN-27 domain answered

While no statistically significant changes were found for youth-reported HRQL, parent-reported psychological well-being increased from baseline to 12 months as measured by KIDSCREEN-27 [F(3,15) = 11.3; p < 0.01]. Parents also reported a statistically significant decrease in youth disability [F(3,15) = 2.7; p = 0.08], whereas youth reports on the WHODAS 2.0 remained stable [F(3,15) = 1.3; p = 0.32]. Youth-reported ACE score showed a statistically significant increase from baseline to 12 months [F(3,15) = 3.4; p = 0.05]. No other statistically significant changes were found.

### Discussion

Response and participation rates were strong and indicate that youth with mental illness are motivated to involve themselves in research that aims to understand mental and psychosocial health even during this vulnerable period in their lives. This finding is reinforced by the strong participation rate among parents. Retention of participants in the study was also acceptable, with dropouts occurring throughout the follow-up. Missing item-level data (i.e., incomplete self-reported questionnaires) was minimal and can be mitigated in larger studies using statistical methods that can accommodate missing data (e.g., multiple imputation, full-information maximum likelihood). The feasibility of the study was confirmed.

The readmission rate in this sample was consistent with recent reports of larger and more diverse samples of youth [22]. In addition, the high proportion of youth with major depressive episode, suicidality, and mental comorbidities was expected [23]. Findings showing improvements in symptoms of mental health, HRQL, and disability were positive, though may be attributed to regression to mean or threshold effects for the measures. For instance, KIDSCREEN-27 scores at the time of hospitalization (i.e., baseline) were some of the lowest reported, even in comparison to other samples of youth with mental illness who were receiving in/outpatient psychiatric services [24]. Given this floor effect, it was likely that HRQL would improve over time in the current study.

It was noteworthy that despite positive findings related to mental and psychosocial health, youth reported increased maltreatment/abuse and household dysfunction in childhood over time. It is plausible that hospitalization represented a time of considerable crisis for youth and that over time, as they reported improved mental health (perhaps with outpatient or community psychiatric aftercare services), they experienced better access to childhood memories and thus reported higher ACE scores. Or, during the follow-up, youth may have experienced events related to maltreatment or family dysfunction, resulting in higher ACE scores. It must also be noted that this finding may be the result of measurement error. Evidence suggests that measures of adverse events in childhood have relatively low reliability in both prospective and retrospective study designs [25].

## Limitations

Limitations to this study warrant consideration. (1) Because the primary aim of this research was to examine the feasibility of recruiting and following youth experiencing their first psychiatric hospitalization, the sample size was small. Thus, this study was likely underpowered to detect changes in mental and psychosocial health over time. (2) Participants were recruited from a single hospital which may limit the generalizability of findings relating to changes in outcomes over time. Selection bias, particularly for the few youth-parent dyads that completed the follow-up, may also impact the reported findings. (3) Testing of multiple mental and psychosocial health outcomes may have increased the probability of detecting a statistically significant result purely based on chance. While there are strong arguments for not adjusting the type I error in the case of multiple testing [26], our use of a more liberal alpha in the context of this feasibility study may have inflated this effect. (4)The COVID-19 pandemic and subsequent countermeasures to reduce transmission have had pervasive effects on individuals, families, and society which may have negatively affected completion rates. Thus, this study may underestimate youth and parent engagement. (5) The observational design of this study prevents any inferences related to causality; that is, there is no evidence to suggest that psychiatric hospitalization resulted in improved reports of mental and psychosocial health in this sample of youth.

## Data Availability

The data collected for this study may be available from the corresponding author upon reasonable request.

## Abbreviations

ACE: Adverse Childhood Experience Questionnaire
MINI-KID: Mini International Neuropsychiatric Interview for Children and Adolescents (MINI-KID)
OCHS-EBS: Ontario Child Health Study Emotional Behavioural Scales
PSS: Perceived Stress Scale
WHODAS: World Health Organization Disability Assessment Schedule
